# Ginseng and its functional components in non-alcoholic fatty liver disease: therapeutic effects and multi-target pharmacological mechanisms

**DOI:** 10.3389/fphar.2025.1540255

**Published:** 2025-04-09

**Authors:** Ping Xiao, Zhaorui Ye, Xiuyan Li, Quansheng Feng, Yue Su

**Affiliations:** School of Basic Medical Sciences, Chengdu University of Traditional Chinese Medicine, Chengdu, China

**Keywords:** ginseng (*Panax ginseng*), non-alcoholic fatty liver disease (NAFLD), lipid metabolism, lipotoxic injury, multi-target pharmacological mechanism

## Abstract

**Background:**

Non-alcoholic fatty liver disease (NAFLD) is a common type of chronic liver disease and its incidence is increasing. Its disease progression is closely related to non-alcoholic steatohepatitis and liver fibrosis. Effective treatment is currently lacking. The traditional Chinese medicine ginseng (*Panax ginseng*) shows unique advantages in NAFLD intervention, but its complex compositional system and molecular mechanism network still need to be systematically analyzed.

**Objective:**

This paper systematically integrates evidence from nearly 20 years of research to elucidate the multi-target pharmacological mechanism of ginseng for the treatment of NAFLD.

**Methods:**

Relevant information was sourced from Pubmed, Web of science, Embase and CNKI databases. Using BioRender and visio to draw biomedical illustrations.

**Results:**

The active ingredients of ginseng contain 2 classes of saponins (tetracyclic triterpene saponins, pentacyclic triterpene saponins and other modified types) and non-saponins. Different cultivation methods, processing techniques and extraction sites have expanded the variety of ginseng constituents and demonstrated different pharmacological activities. Studies have shown that ginseng and its functional components have the ability to regulate lipid metabolism disorders, inflammation, oxidative stress, endoplasmic reticulum stress, insulin resistance, disruption of intestinal flora structure, cell death and senescence. Demonstrates the potential of ginseng for the treatment of NAFLD.

**Conclusion:**

This study reveals for the first time the integrative mechanism of ginseng in the treatment of NAFLD through the tertiary mode of action of “multi-component multi-target multi-pathway”. The multilevel modulatory ability of ginseng provides a new direction for the development of comprehensive therapeutic strategies for NAFLD.

## 1 Introduction

Non-alcoholic fatty liver disease (NAFLD) is characterized by hepatocellular steatosis and fat accumulation without secondary causes of hepatic fat accumulation, such as heavy alcohol consumption, long-term use of lipotropic drugs, or monogenic genetic disorders (Wei et al., 2024). In recent years, NAFLD has become the most common chronic liver disease worldwide. Epidemiologic data show that the global prevalence of NAFLD is as high as 30%, and the prevalence of NAFLD in overweight and obese groups is even as high as 70% (Lou et al., 2024; Quek et al., 2023). Markov modeling predicts that the disease burden of NAFLD-associated cirrhosis and hepatocellular carcinoma will increase by 115%–130% by 2040, leading to serious challenges for healthcare systems worldwide (Devarbhavi et al., 2023). The pathogenesis of NAFLD begins with nonalcoholic fatty liver, which progresses to non-alcoholic steatohepatitis (NASH) in about 20% of patients, driven by oxidative stress, inflammation, and other pathologic factors. Among patients with NASH, approximately 15%–20% progress to cirrhosis within 30–40 years (Li et al., 2024c; Loomba et al., 2021).

Current clinical management strategies for NAFLD are slightly limited. The common treatment of NAFLD is aimed primarily at alleviating the associated metabolic comorbidities and cardiovascular disease. For example, reducing hyperlipidemia, insulin resistance (IR) and hyperglycemia associated with NAFLD, rather than directly treating the disease itself (Xu et al., 2022). NAFLD patients who do not develop NASH or liver fibrosis have a good prognosis, and their disease can even be reversed (Loomba et al., 2021). First-line therapies are based on lifestyle interventions, but patient compliance is poor, resulting in a high risk of disease progression (Younossi et al., 2023). Pharmacologic therapy is primarily directed at patients who have progressed to NASH and liver fibrosis, but approved drugs (vitamin E and pioglitazone) have limited efficacy. Clinical studies have shown that pioglitazone therapy for NASH has a 47% remission rate, but does not improve fibrosis and may cause weight gain and fluid retention (Paternostro and Trauner, 2022). Although novel agents “glucagon-like peptide-1 receptor agonists and farnesoid X receptor (FXR) agonists” have entered clinical trials, their long-term safety, cost-effectiveness, and efficacy in advanced fibrosis remain controversial (Newsome et al., 2021; Younossi et al., 2022). End-stage patients are dependent on liver transplantation, but donor shortages and postoperative immunosuppressive complications result in 5-year survival rates of only 65% (Terrault et al., 2023). Therefore, the development of novel therapeutic strategies with multi-targeted effects, high safety profile and the ability to block early disease progression is urgent.

**FIGURE 1:**
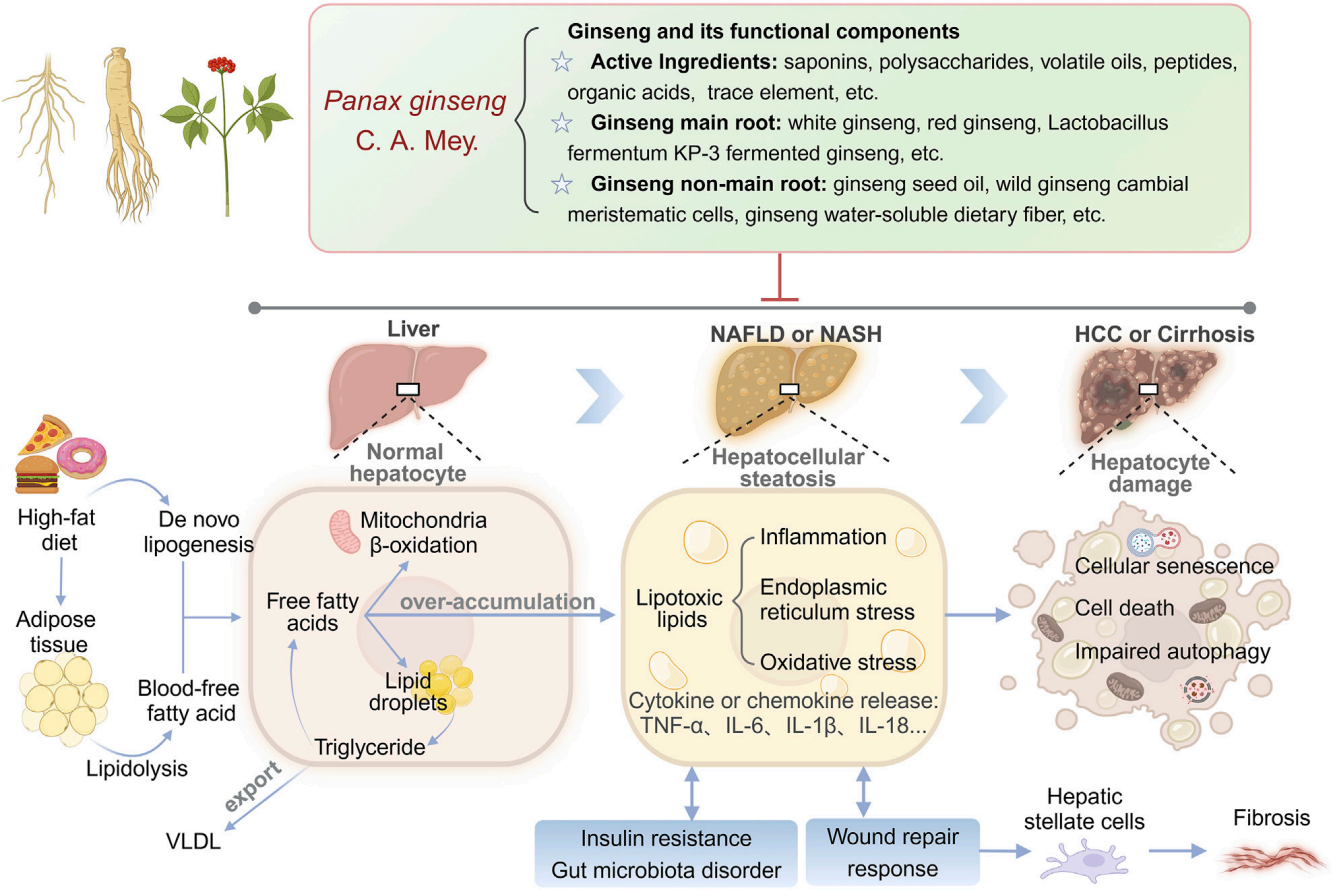
Multi-target mechanisms of ginseng in NAFLD/NASH: from lipid metabolism to hepatocyte damage.

Compared with synthetic drugs, traditional Chinese medicine (TCM), as a complementary therapy for liver diseases, has become a new direction for NAFLD drug development due to its wide range of efficacy and multi-component-multi-target properties (Li et al., 2024b; Liu et al., 2022; Zhang and Feng, 2022). Complementary herbal therapies are mostly used clinically for the comprehensive regulation of NAFLD and NASH, among which the hepatoprotective effects of ginseng and its compounds have attracted much attention (Chen et al., 2021). Clinical studies have confirmed that ginseng and its functional components improve metabolic disorders through the following mechanisms: (1) Improve blood glucose and lipid levels by regulating the phosphatidylinositol-3-kinase/protein kinase B (PI3K/AKT) signaling pathway. (2) Inhibiting inflammation-related signaling pathways “nuclear factor kappa-B (NF-κB), mitogen-activated protein kinases, janus kinase 2/signal transducer and activator of transcription (STAT) 5” to reduce inflammation. (3) Scavenges free radicals and reduces cellular damage from oxidative stress (Zhou et al., 2023). Notably, ginseng has shown more comprehensive efficacy than a single synthetic drug in animal models of NAFLD, providing simultaneous improvement in steatosis, IR, and hepatic fibrosis, while demonstrating low toxicity and conferring many benefits (Wang et al., 2021; Yang K. et al., 2023). In this paper, for the first time, we systematically elucidate the multidimensional mechanism of ginseng intervention in NAFLD by integrating nearly 20 years of experimental research evidence, focusing on resolving its key target networks in lipid metabolism regulation, lipotoxic damage repair, cell death and senescence reversal, IR and gut microbiota remodeling (Figure 1). By revealing the component-target-pathway interaction patterns, this study aims to promote the progress of traditional medicine modernization research and provide a theoretical basis for the development of innovative strategies based on the multi-targets of TCM for the treatment of NAFLD.

## 2 Pathogenesis of NAFLD

The pathogenesis of NAFLD is complex. The early “two-hit” hypothesis was considered the central model to explain the progression of NAFLD (Buzzetti et al., 2016). This hypothesis suggests that the first hit is triggered by excessive hepatic lipid deposition, while the second hit is driven by secondary damage, such as inflammation and endoplasmic reticulum stress (ERS), which ultimately leads to NASH and fibrosis. However, with the deepening of the research, the limitations of the “two-hit” model have gradually appeared, and its assumption of linear phasing is difficult to explain the heterogeneity of NAFLD, multi-organ interactions, and the systemic effects of metabolic syndrome. In this context, scholars have proposed the “multiple-hit” hypothesis, which emphasizes that NAFLD is the result of synergistic or superimposed effects of multiple pathological factors in space and time (Buzzetti et al., 2016; Wang et al., 2023). Unlike the “two-hit” model, the “multiple-hit” hypothesis is no longer limited to localized events in the liver, but incorporates extra-hepatic factors (e.g., adipose tissue dysfunction, intestinal flora disruption) into the core mechanistic framework. Excessive lipid accumulation also promotes the production of lipotoxic substances, which in turn activate oxidative stress (OS), inflammation and ERS processes, leading to secondary liver damage and further inducing cell death or senescence (Mahlapuu et al., 2022; Xu et al., 2020). If pathological factors persist, the liver will undergo repeated injury and repair processes and activate hepatic stellate cells, leading to fibroplasia and even cirrhosis (Taru et al., 2024). Inflammatory cytokines, adipokines, dietary, genetic and environmental factors have important effects on IR and gut microbiota homeostasis, and conversely, IR and gut microbiota disorders exacerbate imbalance of lipid metabolism (Chen and Vitetta, 2020; Fujii et al., 2020; Khan et al., 2019; Malesza et al., 2021). Complex pathological mechanisms mutually drive the development of aberrant lipid metabolism in the liver, including excessive nutrient intake, lipotoxicity, activation of the hepatic immune system, OS, ERS, cell death and senescence, IR, disturbances of the gut microbiota, and genetic and epigenetic factors, which are the key risk factors for the development of NAFLD (Buzzetti et al., 2016; Friedman et al., 2018; Huby and Gautier, 2022). Some studies have been reported to demonstrate that if hepatocellular steatosis can be reversed by early intervention through metabolic pathways, the risk causative factors and metabolism-related complications of NAFLD can be mitigated (Loomba et al., 2021). Therefore, we should develop more potential therapeutic approaches to prevent and treat NAFLD.

## 3 Traditional and modern cognition of ginseng

Ginseng (Latin name: *Panax ginseng* C.A. Mey.) is a perennial herb in the family araliaceae and is widely used worldwide (Ratan et al., 2021). It is widely known as “the king of herbs, the head of all medicines” in East Asian traditional medicine such as China, Korea and Japan (Potenza et al., 2023). In the famous book *Chinese Pharmacopoeia (2020 Edition)*, there is an exhaustive account of ginseng. It has been used in China for more than 2000 years (Li G. et al., 2024). In modern applications of TCM, ginseng is often used to treat lack of power, shortness of breath, palpitations and insomnia, haemorrhage and life-threatening conditions (Li X. et al., 2022; Ma et al., 2023). It exhibits unique energy-boosting properties that fit the clinical manifestations of fatigue, weakness and abdominal distension and discomfort that are common in NAFLD patients (Lu et al., 2021).

Ginseng has a complex composition including saponins, polysaccharides, volatile oils, peptides, organic acids and trace elements, among others (Li X. et al., 2022; Piao et al., 2020). The pharmacological effects of these components have significant target specificity, and the active ingredients commonly used in current experiments can be broadly categorized into the following 2 groups. (1) Ginsenosides are the core active ingredients of ginseng. According to the structure of the glycosides, they can be categorized into tetracyclic triterpene saponins, pentacyclic triterpene saponins and other modified types. The main saponin currently used in the treatment of NAFLD is a tetracyclic triterpene saponin. It includes protopanaxadiol (PPD), protopanaxatriol (PPT) and PPD/PPT-type saponin derivatives (Table 1). (2) Non-saponin components, such as the polyacetylene alcohol compounds: panaxydol. The above multicomponents target multiple pathological aspects such as lipid metabolism, inflammation, OS and fibrosis through synergistic effects, providing a material basis for the multi-targeting properties of ginseng in the treatment of NAFLD.

**TABLE 1:** Classification and representative components of tetracyclic triterpenoid saponins used in NAFLD.

Aglycone structures	Representative components
Protopanaxadiol (PPD)	Rb_1_, Rb_2_, Rc, Rd, Rg_3_, Rh_2_, etc.
Protopanaxatriol (PPT)	Rg_1_, Rg_2_, Re, Rf, Rh_1_, 20(S)-PPT, etc.
PPD/PPT-type saponin derivatives	C17-side-chain variant (Rg_3_, Rk_3_, etc.), deglycosyl-derived saponins (CK, Mc_1_, F_2_, etc.)

The functional components of ginseng are closely related to the cultivation method, processing technology and extraction site. Those sown in the forest and grown naturally are called forest ginseng, and those harvested after 5-6 years of artificial cultivation are commonly called garden ginseng (Li G. et al., 2024). The long time of ginseng cultivation and the small number of wild plants often lead to expensive prices and limited resources (Li G. et al., 2024). Therefore, researchers have expanded its medicinal value through processing optimization, microbial fermentation technology and multi-site development (Wang et al., 2025). According to the sources and preparation methods, they can be categorized into the following 2 groups. (1) The active ingredient profile is altered by physical or biological transformation using the main root of ginseng as the main body. For example, fresh ginseng washed and dried in the sun is known as sun-dried ginseng, which will retain native saponin components to a greater extent and has the effect of improving lipid metabolism disorders in NAFLD (Li et al., 2022c). Fresh ginseng that has been steamed and dried is called red ginseng (RG), which promotes the conversion of native saponins (Rb_1_) into rare saponins (Rg_3_, Rh_2_ and CK). It has been shown to promote reverse cholesterol transport and anti-inflammatory effects (Li et al., 2022c). Microbial fermentation (*Lactobacillus* fermentum KP-3 and Monascus ruber) increased the content of secondary ginsenosides, small-molecule peptides, and polysaccharides (Nan et al., 2018; Zhao et al., 2021). Cordyceps militaris fermentation enhances the content of rare ginsenoside (Rg_3_) (Li et al., 2020). In addition, some studies have used modern techniques to efficiently enrich active ingredients from ginseng roots. For example, targeted enzymatic cleavage of ginsenoside glycosyl groups to generate highly bioavailable glycosides (GBCK25) (Choi N. et al., 2019). The active ingredients generated have the ability to reduce fatty apoptosis and cholesterol synthesis in hepatocytes. (2) Exploiting the pharmacological potential of non-primary root parts of the ginseng plant, including cold-pressed extraction of ginseng seed oil (GSO), *in vitro* cultivation of wild ginseng meristematic tissue cells, and extraction of water-soluble dietary fibers from ginseng whiskers (Hua et al., 2021; Kim et al., 2018; Lee et al., 2016). These processed ginsengs have a wider range of pharmacological effects, and we summarize the above ginsengs, concoctions and their characteristic extracts into a table (Table 2).

**TABLE 2:** Effects of processing parts and processing methods on the composition and mechanism of action of ginseng.

Category	Name	Processing technology	Representative characteristic ingredients	Representative mechanisms of action	Ref.
Ginseng main root	Sun-dried ginseng	Washed and dried	Primary saponins predominant (Rg_1_, Re, etc.)	Improve lipid metabolism disorders; strong antioxidant capacity	Chung et al. (2016)
	RG	Steam-dried	Higher content of rare saponins (Rg_3_, Rh_2_, CK, etc.)	Promotes reverse cholesterol transport; anti-inflammatory	Kwak et al. (2024)
	LFG	*Lactobacillus* fermentum KP-3 fermentation	Rg_2_, Rg_3_, Rh_1_, Rh_2_, F_2_, Ro, small molecule peptide, etc.	Improve lipid metabolism disorders; anti-inflammatory	Nan et al. (2018)
	MFG	Monascus ruber fermentation	Rg_1_, Re, Rc, Rd, polysaccharides, etc.	Increases bile acid excretion; regulates intestinal flora	Zhao et al. (2021)
	FRG	Cordyceps militaris fermentation	Rg_3_, Rd, etc.	Modulation of immune functions	Li et al. (2020)
	FG	*Saccharomyces* servazzii GB-07 strain and pectinase fermentation	GBCK25	Reduction of OS; reduction of hepatocyte steatosis apoptosis	Choi et al. (2019a)
Ginseng non-main root	GSO	Cold-pressed ginseng seed oil	Oleic acid, linoleic acid, palmitic acid, phytosterols, etc.	Inhibits *de novo* lipogenesis; improves IR	Kim et al. (2018)
	WGCM	*In vitro* culture of wild ginseng meristematic tissue cells	Rg_3_, Rh_2_, Rk_1_, Rg_5_, etc.	Improves mitochondrial function; reduces OS	Lee et al. (2016)
	GWDF	Soluble dietary fiber extracted from lateral root of ginseng	Glucose, galactose, mannose, etc.	Regulates glucose-fat metabolism; promotes colon health; regulates appetite and energy balance	Hua et al. (2021)

Natural products as supplementation agents are beneficial in promoting glycolipid metabolism and improving hepatocellular steatosis to halt the progression of NAFLD (Cao et al., 2023; Leng et al., 2021). Compared with other traditional medicinal herbs, ginseng exhibits a variety of irreplaceable multidimensional advantages that make it unique in NAFLD intervention. For example, multiple components of ginseng can simultaneously target multiple pathways such as AMP-activated protein kinase (AMPK), peroxisome proliferator-activated receptor (PPAR) and sterol-regulatory element binding protein (SREBP) (Mi et al., 2024). The lipid metabolism pathology network of NAFLD can be improved through the “one drug, multiple effects” model. Mc_1_ can improve apoptosis and insulin sensitivity, realizing the comprehensive improvement of NAFLD across pathological links, which has more clinical potentials than the herbs with a single mechanism of action (Roh et al., 2020). In addition, ginseng has been used for thousands of years without any serious liver injury, and modern toxicology has confirmed that it has no significant side effects at therapeutic doses (Park et al., 2022). Therefore, ginseng is not only a traditional tonic medicine, but also a multi-target liver metabolism regulator verified by modern science.

## 4 Pharmacological effects and molecular mechanisms of ginseng against NAFLD

Laboratory studies on ginseng anti-NAFLD have shown that ginseng and its functional components can block multiple underlying pathological mechanisms, including regulating disorders of lipid metabolism in the body, reducing hepatocellular inflammation, ERS, OS, IR, intestinal flora disorders, cellular senescence and death. During the onset of NAFLD, ginseng regulates a variety of hepatic functions such as synthesis, storage, catabolism, transport and secretion, thus exerting a protective effect on the normal structure and function of the liver. A detailed discussion is provided below to facilitate the reader’s access to relevant experimental studies, and we use BioRender and visio to draw biomedical illustrations.

### 4.1 Lipid metabolism

Excessive accumulation of lipids in hepatocytes is the earliest and most common response to NAFLD (Ebert et al., 2023). After years of research, ginseng and its functional components have been found to directly regulate multiple pathways of lipid metabolism, including the regulation of lipid uptake and transport, increase of lipolysis and decrease of lipid synthesis, etc. The following is a systematic and comprehensive review of the role of ginseng and its functional components in targeting the above lipid metabolism pathways (Table 3).

**TABLE 3:** Summary of the mechanisms by which ginseng improves lipid metabolism.

Ginseng	Models	Lipid metabolism	Ref.
20(S)-PPT	Mouse PHs, HepG2 cells	TG↓, LXRα↓, SREBP-1c↓, FAS↓, SCD-1↓	Oh et al. (2015)
Aged ginseng	C57BL/6N male mice	TG↓, TC↓, HDL-C↑, FAS↓, adiponectin↑, leptin resistance↓, appetite↓	Chung et al. (2016)
CK	HuH7 cells	TG↓, AMPK↑, ACC↓, LDs↓, PPAR-α↑, ACOX-1↑	Kim et al. (2013)
FG	male ICR mice	TG↓, LDL-C↓, microsomal triglyceride transfer protein↓, apolipoprotein A4↑	Park et al. (2018)
FRG	C57BL/6N male mice, mouse PHs	LDs↓, ACC↓, FAS↓, SREBP-1c↓, PPAR-α↑, CPT-1↑, ACOX-1↑, FA translocase↓, acyl-CoA synthetase long-chain↓	Choi et al. (2019b)
GBCK25	C57BL/6 male mice, alpha mouse liver 12 cell line, mouse KCs, murine monocyte/macrophage cell line RAW264.7	TG↓, TC↓, FAS↓, ACC-1↓	Choi et al. (2019a)
GDS	C57BL/6 male mice, HepG2 cells	TG↓, TC↓, LDL↓, HDL-C↑, fat-specific protein 27↓, AMPK↑, SREBP-1c↓, ACC↓, FAS↓, CPT-1↑, CPT-2↑, PPAR-α↑	Mi et al. (2024)
GF_2_	C57BL/6J male and LXRα deficient mice, mouse BMDMs, mouse PHs	TG↓, LXRα↓, FAS↓, SREBP-1↓	Kim et al. (2018)
Ginsenosides	C57BL/6J male mice	TG↓, TC↓, LDL-C↓, LDL-C/HDL-C↓, SREBP-1c↓, FAS↓, ACC-1↓, CPT-1α↑, leptin resistance↓	Liang et al. (2021)
GSO	C57BL/6J mice, HepG2 cells, rat PHs	TG↓, TC↓, LDL-C↓, HDL-C↑, SIRT1↑, PPAR-α↑, PGC-1α↑, CPT-1α↑, SREBP-1↓, ChREBP↓	Kim et al. (2018)
GWDF	male SD rats	TG↓, ghrelin↑, glucagon-like peptide 1↑, peptide YY↑, cholecystokinin↑	Hua et al. (2021)
LFG	C57BL/6N male mice	TG↓, TC↓, LDL↓	Nan et al. (2018)
Mc_1_	C57BL/6 male mice, HepG2 cells	TG↓, SREBP-1c↓, FAS↓, ACC↓	Roh et al. (2020)
MFG	SD male rats	TC↓, LDL-C↓, SREBP-2↓, HMGCR↓, FXRα↓, CYP7A1↑, fecal total bile acids↑	Zhao et al. (2021)
Mixture Rh_1_ and Rg_2_	C57BL/6 male mice, mouse KCs, mouse PHs, mouse hepatic stellate cells	TC↓, LDs↓, FAS↓, SREBP-1c↓, ChREBP↓, PPAR-α↑, CPT-1α↑	Wang et al. (2021)
Non-fermented ginseng	male ICR mice	TG↓, FFAs↓, microsomal triglyceride transfer protein↓, SREBP-1↓, PPAR-α↑, SCD-1↓	Park et al. (2018)
Rb_1_	Zebrafish, male long-evans rats, rat PHs, C57BL/6J male mice, mouse 3T3-L1 fibroblast cells, HepG2 cells	TG↓, TC↓, LDs↓, SREBP-2↓, LDL receptor↓, HMGCR↑, CYP7A1↑, AMPK↑, adiponectin↑, ACC↓, SREBP-1c↓, FAS↓, SCD-1↓, PGC-1α↑, PPAR-α↑, CPT-1↑, ACOX-1↑	Li et al. (2020), Li et al. (2022d), Meng et al. (2023), Shen et al. (2013)
Rb_2_	C57BL/KsJ-lepdb (db/db) mice, HepG2 cells	TG↓, TC↓	Huang et al. (2017)
Rc	C57BL/6J mice, mouse PHs	TG↓, TC↓, SREBP-1c↓, FAS↓, CPT-1α↑, CPT-1β↑, SIRT6↑, PPAR-α↑	Yang et al. (2023b)
Rd	C57BL/6J mice, mouse PHs	TG↓, TC↓, SREBP-1c↓, FAS↓, ACC↓, CPT-1α↑, CPT-1β↑, CPT-2↑, SIRT6↑, PPAR-α↑	Cui et al. (2023)
Rf	HepG2 cells	DNMT3L gene, ANXA2 genne	Chen et al. (2022)
RG	thoroughbred riding horses, otsuka long-evans tokushima fatty rats, C57BL/6 mice, mouse PHs and KCs	TG↓, FFAs↓, LXRα↓, LXRβ↓, FAS↓, ACC-1↓, HDL-C↑	Hong et al. (2013), Jeong et al. (2018), Kwak et al. (2024)
Rg_1_	HepG2 cells, SD male rats, C57BL/6J mice	TG↓, TC↓, FFAs↓, LDL↓, LDL-C↓, HDL-C↑, acyl-CoA synthetase↑, CPT-1↑, ACOX-1↑, AMPK↑, ACC-1↑, SREBP-1c↓, FAS↓, PPAR-α↑	Hou et al. (2022), Xiao et al. (2019), Xu et al. (2018), Xuan et al. (2015)
Rg_3_	C57BL/6 male mice, mouse 3T3-L1pre-adipocyte cells, HepG2 cells	TG↓, TC↓, FFAs↓, AMPK↑, ACC-1↓, SREBP-2↓, HMGCR↓, STAT5↓, PPAR-γ↓, FA binding protein 4↓, SCD-1↓	Lee et al. (2017), Lee et al. (2012)
Rk_3_	C57BL/6J male mice, HepG2 cells, LX2 cell lines	PI3K/AKT↑, TG↓, TC↓, LDL↓	Guo et al. (2023)
WGCM	C57BL/6 male mice	TG↓, TC↓, SREBP-1↓, ACC↓, FAS↓, ChREBP↓, PPAR-α↑, CPT-1α↑	Lee et al. (2016)

Note: BMDMs, bone marrow-derived macrophages; KCs, kupffer cells; PHs, primary hepatocytes; SD, sprague dawley. ↓: inactivate or decrease; ↑: activate or increase.

#### 4.1.1 Regulation of lipid uptake and transportation

**FIGURE 2:**
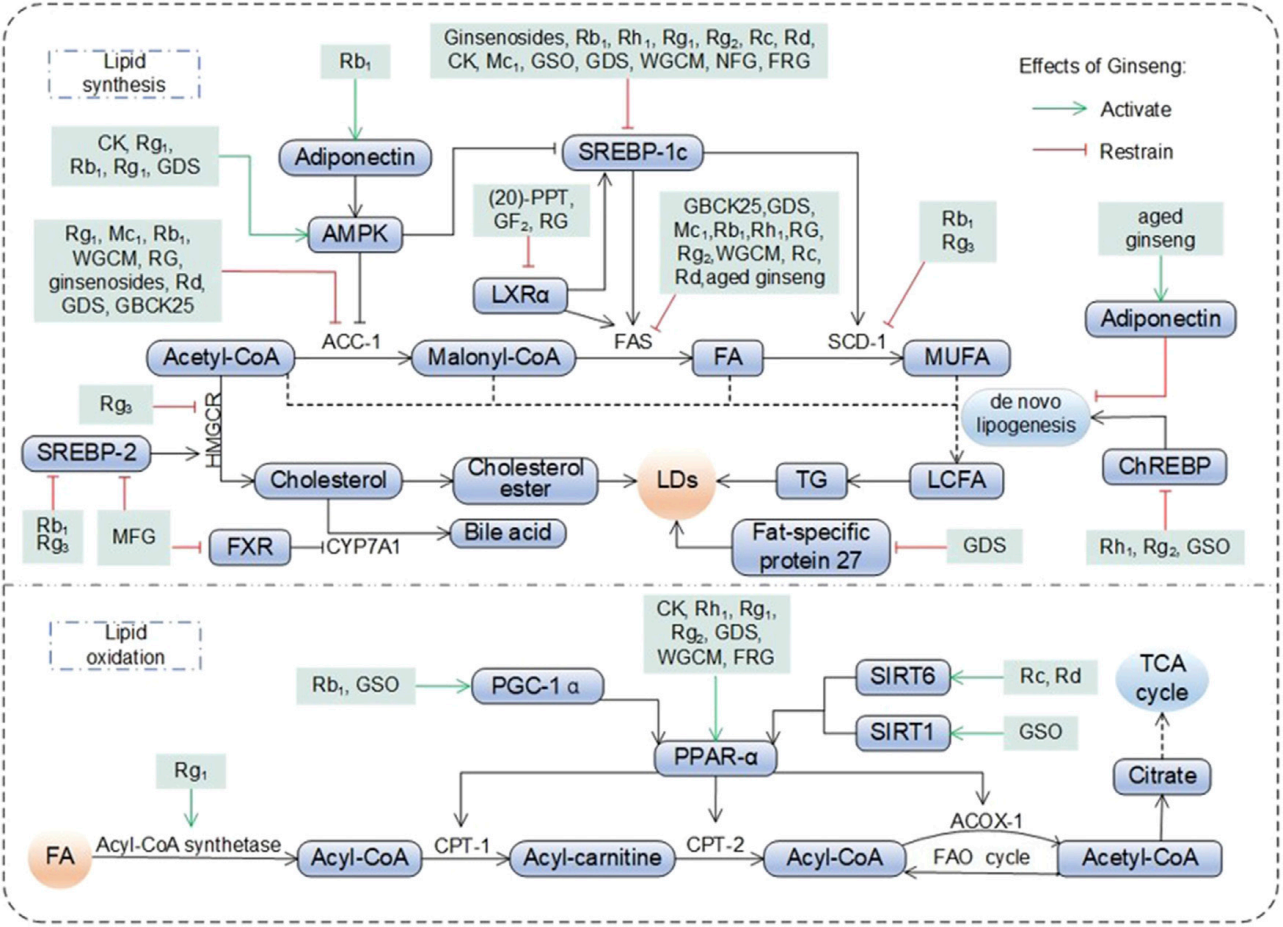
Pharmacological effects and molecular mechanisms of ginseng in regulating lipid metabolism. Fatty acids (FA) entering hepatocytes are broken down into acetyl-CoA by acetyl-CoA synthetase, which enters the mitochondria and participates in the fatty acid oxidation cycle (FAO cycle) or the tricarboxylic acid cycle (TCA cycle) under the promotion of various active substances. Among them, acetyl-CoA also participates in lipid synthesis in the organism, generating long chain fatty acid (LCFA, the process is also known as *de novo* lipogenesis) under the promotion of AMP-activated protein kinase (AMPK), acetyl-coenzyme A carboxylase 1 (ACC-1), liver X receptor a (LXRa), sterol-regulatory element binding protein (SREBP) 1c, fatty acid synthase (FAS) and stearoyl-coenzyme A desaturase 1 (SCD-1), on the one hand, lipid droplets (LDs) was generated thereafter. On the other hand, cholesterol was generated under the promotion of SREBP-2 and 3-hydroxy-3-methylglutaryl- coenzyme A reductase (HMGCR), which in turn synthesizes LDs, or is broken down by farnesoid X Receptor (FXR) and CYP7A1 into bile acid and excreted. ACOX-1: acyl-coenzyme A oxidase 1; ChREBP: carbohydrate responsive element-binding protein; CK: compound K; CPT: carnitine palmityl transferase; GDS: ginseng diol saponin; GSO: ginseng seed oil; MFG: monascus ruber fermented ginseng: MUFA: monounsaturated fatty acid; NFG: nonfermented ginseng: PGC-1a: peroxisome proliferators-activated receptor y co-activator a; PPAR-a: peroxisome proliferator-activated receptor a; PPT: protopanaxatriol; SIRT: sirtuins; TG: triglyceride; WGCM: wild ginseng cambial meristematic cells.

As the hub of systemic metabolism, the liver is prone to lipid metabolism disorders in hyperlipidemic and obese states, which directly promote the progression of NAFLD (Mato et al., 2019). Studies have shown that ginseng improves lipid homeostasis through the following mechanisms (Figure 2). Significantly reduces triglyceride (TG), total cholesterol (TC), free fatty acids (FFAs), lipid droplets (LDs), low density lipoprotein (LDL), low density lipoprotein cholesterol (LDL-C), LDL-C to high density lipoprotein cholesterol (HDL-C) ratio (LDL-C/HDL-C) in the liver and serum (Guo et al., 2023; Liang et al., 2021; Meng et al., 2023; Xu et al., 2018). In addition evidence of pathologic improvement showed that model animals raised with ginseng had lighter liver weights and body weights, more aligned hepatocytes, fewer vacuoles, and less steatosis (Meng et al., 2023). Analysis of FFAs using molecular networking showed that RG fed thoroughbred riding horses reduced pro-inflammatory FFAs (C12:0, dodecanoic acid; C14:0, myristric acid; C18:1, oleic acid; C18:2, linoleic acid) (Kwak et al., 2024). The above studies demonstrated some intuitive manifestations of the hypolipidemic properties of ginseng and its functional components.

Aged ginseng reduced appetite and decreased the body’s lipid intake at the source (Chung et al., 2016). Ginseng water-soluble dietary fiber (GWDF) upregulated glucagon-like peptide 1, peptide YY, cholecystokinin and serum gastric hunger hormone levels. It increased satiety, promoted digestion, delayed gastric emptying, decreased food intake and affected glucolipid metabolism in male SD rats (Hua et al., 2021). Aged ginseng and ginsenosides acted as modulators and significantly reversed abnormally high leptin levels. Restores leptin’s appetite-suppressing function by reducing leptin resistance (Chung et al., 2016; Liang et al., 2021).

HDL-C and apolipoprotein A4 (apoA4) have a role in transporting cholesterol from peripheral tissues to the liver for removal. Ginseng and its functional components promotes reverse cholesterol transport into the liver and accelerates cholesterol breakdown and excretion by up-regulating HDL-C and apoA4 (Kim et al., 2018; Park et al., 2018; Xuan et al., 2015). Microsomal triglyceride transfer protein is a lipid transfer protein necessary for the synthesis of very low density lipoprotein in hepatocytes, and its synthesis is upregulated in response to increased TG. Fermented ginseng (FG) and non-fermented ginseng reduces very low density lipoprotein secretion and alleviates hepatic TG accumulation by inhibiting MTP expression (Park et al., 2018). Monascus ruber fermented ginseng (MFG) inhibits FXR receptor, increases cholesterol 7α-hydroxylase (CYP7A1) expression, and promotes bile acid synthesis. Rb_1_ directly promotes CYP7A1 activity, accelerating the conversion of cholesterol to bile acids and promoting cholesterol excretion (Li et al., 2020; Zhao et al., 2021).

#### 4.1.2 Regulation of lipid synthesis and decomposition

On the one hand, hepatic nascent fatty acid (FA) are esterified to TG and will be stored as LDs in hepatocytes or secreted in other forms into the bloodstream, and on the other hand, it is directly metabolised by the β-oxidation pathway (Badmus et al., 2022). Therefore, an increase in nutrients delivered to the liver and FA synthesis, a decrease in FA oxidation and lipid output will result in an excessive accumulation of fat in the liver (Badmus et al., 2022). Ginseng and its functional components are a potent modulator of lipid metabolism (Figure 2).

Ginseng and its functional components plays a key role in reducing FA synthesis by directly inhibiting the expression of lipid synthesis genes such as SREBP-1 gene (isoforms SREBP-1a and SREBP-1c), fatty acid synthase (FAS) gene, stearoyl-coenzyme A desaturase-1 (SCD-1) gene and acetyl-coenzyme A carboxylase (ACC, ACC-1 and ACC-2 subtypes) 1 (Choi N. et al., 2019; Park et al., 2018; Wang et al., 2021). AMPK is a key sensor of cellular energy status. Rg_1_ significantly increased AMPK phosphorylation and restored hepatic lipid homeostasis (Xiao et al., 2019). CK and Rg_3_ inhibited downstream ACC-1 expression and reduced hepatic lipogenesis by activating AMPK phosphorylation (Kim et al., 2013; Lee et al., 2012). In addition, Rb_1_ and ginseng diol saponin (GDS) significantly inhibited SREBP-1c activity upon activation of AMPK, reducing the activation of downstream targets FAS and SCD-1 (Mi et al., 2024; Shen et al., 2013). GSO, Rh_1_ and Rg_2_ block *de novo* lipogenesis by directly blocking carbohydrate responsive element-binding protein (ChREBP) nuclear translocation and the synthesis of ACC, SCD-1, and FAS. In addition, wild ginseng cambial meristematic cells (WGCM) inhibits DNL by directly reducing the synthesis of the key enzymes ACC, SCD-1, and FAS (Kim et al., 2018; Lee et al., 2016; Wang et al., 2021). Lipocalin is an insulin-sensitive adipocyte-specific cytokine that inhibits *de novo* lipogenesis. Aged ginseng inhibited *de novo* lipogenesis and delayed NAFLD progression by upregulating lipocalin expression (Chung et al., 2016).

RG reduced the expression of hepatic lipid metabolism-related factors liver X receptors (LXR) α and LXRβ. This process regulates intracellular cholesterol and lipid homeostasis and reduces pro-inflammatory factor production. (Jeong et al., 2018). 20(S)-PPT and GF_2_ regulated the LXRα by inhibiting the transcription of the adipogenic genes SREBP-1c and FAS to reduce adipogenesis (Kim K. et al., 2024; Oh et al., 2015). Rb_1_, MFG and Rg_3_ blocked the SREBP-2/3-hydroxy-3-methylglutaryl-coenzyme A reductase (HMGCR) pathway, while Rg_3_ directly inhibited the expression of the downstream gene HMGCR (Lee et al., 2012; Li et al., 2020; Zhao et al., 2021). The above processes contribute to the inactivation of key pathways of cholesterol synthesis and reduce cholesterol biosynthesis. GDS targets fat-specific protein, a key protein in lipid droplet synthesis, to reduce its expression and significantly decrease the area of hepatocyte LDs, thereby reducing fat storage (Mi et al., 2024).

During FA oxidation, ginseng and its functional components maintains hepatic lipid homeostasis by stimulating the transcription of PPAR α and PPAR-γ response genes (Liang et al., 2021; Xuan et al., 2015). In addition ginseng and its functional components also synergizes with carnitine palmitoyl transferase (CPT) to accomplish FA oxidation. Examples include the transport and oxidation of acyl-CoA from the cytoplasm to the mitochondrial matrix, and FA oxidation processes involving acyl-CoA synthetase and ester oxygenase (Choi S. Y. et al., 2019; Kim et al., 2013; Mi et al., 2024). One study further found that GSO, Rc and Rd promote PPAR-α-mediated FA oxidation by increasing the expression of sirtuins (SIRT) 1 or SIRT6 proteins (Cui et al., 2023; Kim et al., 2018; Yang Z. et al., 2023). GSO and Rb_1_ synergistically regulated PPAR-α-mediated FAO by activating peroxisome proliferators-activated receptor γ co-activator α (PGC-1α) (Kim et al., 2018; Shen et al., 2013). STAT5 promotes the binding of PPAR-γ to PPAR response elements and regulates downstream genes related to adipogenesis, lipid metabolism and glucose homeostasis. However, Rg_3_ blocked STAT5 and PPAR-γ associated target adipogenesis and inhibited the adipogenic process in 3T3-L1 cells (Lee et al., 2017). Fermentation of red ginseng with C. militaris (FRG) reduced adipogenesis and lipid uptake by decreasing the expression of acyl-CoA synthetase long-chain and FA translocase (Choi S. Y. et al., 2019). Bioinformatic experiments and cellular experiments validated that Rf downregulated the methylation level of the DNMT3L gene and reversed the aberrant expression of the adipogenesis-related gene ANXA2 (Chen et al., 2022).

### 4.2 Lipotoxic injury

**FIGURE 3:**
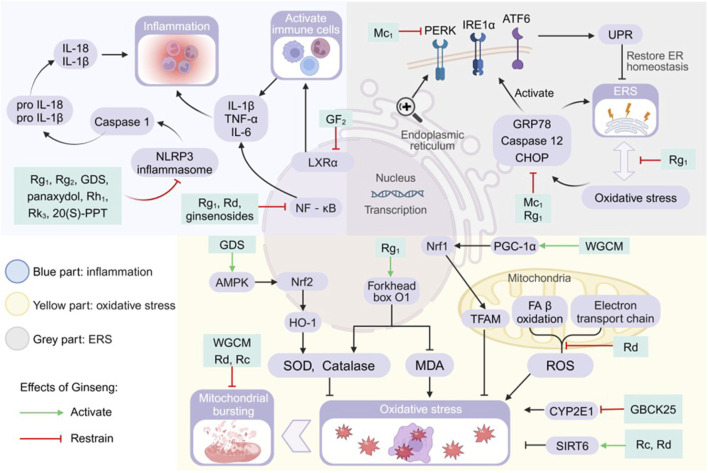
Pharmacological effects and molecular mechanisms of ginseng in ameliorating lipotoxic injury. Blue part: activation of nod-like receptor thermal protein domain associated protein 3 (NLRP3) inflammasome, nuclear factor kappa-B (NF-kB) pathway, and liver X receptor a (LXRa) all promote the release of inflammatory factors or the activation of immune cells, but ginseng and its characteristic extracts have the effect of inhibiting the activation of the above substances or pathways. Gray part: increased glucose-regulated protein78 (GRP78), cysteine-containing aspartate-specific proteases (Caspase) 12 and CCAAT/enhancer binding protein (C/EBP) homologous protein (CHOP) promote endoplasmic reticulum stress (ERS), and they also promote unfolded protein response (UPR) by activating the signaling cascade promoters PRKR-like ER kinase (PERK), inositol-requiring enzyme 1a (IRE1a) and activating transcription factor 6 (ATF6). Ginseng has a mitigating effect on ERS and UPR. Yellow part: the AMP-activated protein kinase/nuclear factor-erythroid 2 related factor 2/heme oxygenase 1 (AMPK/Nrf2/HO-1) pathway, forkhead box 01 genes, mitochondrial transcription factor A (TFAM) and sirtuins (SIRT) 6 promote the production of antioxidants or directly inhibit oxidative stress, and ginseng strengthens their efficacy. Mitochondrial ETC and fatty acid (FA) ẞ oxidation generated reactive oxygen species (ROS) and cytochrome P450 2E1 (CYP2E1) both promoted oxidative stress, and ginseng inhibited their activities. GDS: ginseng diol saponin; IL: interleukin; MDA: malondialdehyde; Nrf1: nuclear respiratory factor 1; PGC-1a: peroxisome proliferators-activated receptor y co-activator a; 20(S)-PPT: 20(S)-protopanaxatriol; SOD: superoxide dismutase; TNF-a: tumor necrosis factor a; WGCM:wild ginseng cambial meristematic cells.

The liver converts excess FA to TG for storage in an early adaptive protective response. If FAs are continuously supplied in excess or their processing is impaired, they become substrates for the production of lipotoxic lipids and activate a range of pathological response mechanisms, such as inflammation, ERS and OS, causing hepatocellular damage and accelerating the progression of NAFLD (Geng et al., 2021). Not only do various pathophysiological mechanisms play an important role in the development of NAFLD, but there are complex interactions between the mechanisms themselves. Lipotoxic substances induce hepatocyte stress, injury and even death, leading to chronic inflammatory response and generation of large amounts of reactive oxygen species (ROS) causing OS in hepatocytes (Peiseler et al., 2022). OS can further exacerbate inflammation and induce the endoplasmic reticulum to produce a large number of error proteins, activating the ERS and promoting the progression of NAFLD to cirrhosis and hepatocellular carcinoma (Peiseler et al., 2022). Preventing the production of lipotoxic substances will be the key to treatment. Modern medical research has provided a range of evidence at both the cellular and animal levels to support the idea that inhibition of the process of lipotoxic damage by ginseng and its functional components may be a key target for amelioration of NAFLD, suggesting the potential of ginseng and its functional components as novel therapeutic agents for the prevention and treatment of NAFLD (Table 4; Figure 3).

**TABLE 4:** Summary of the mechanisms by which ginseng ameliorates lipotoxic injury.

Ginseng	Models	Lipotoxic injury	Ref.
20(S)-PPT	C57BL/6 male mice, mouse BMDMs, mouse PHs and KCs, human peripheral blood mononuclear cells	ALT↓, AST↓, NLRP3↓, IL-1β↓, TNF-α↓	Lu et al. (2023)
Aged ginseng	C57BL/6N male mice	ALT↓, AST↓, TNF-α↓	Chung et al. (2016)
FRG	C57BL/6N male mice, mouse PHs	ALT↓, AST↓, IL-1β↓, IL-6↓, TNF-α↓, CCL2↓, CCL5↓, inducible nitric oxide sythase↓, IL-10↑, CD163↑	Choi et al. (2019b)
GBCK25	C57BL/6 male mice, alpha mouse liver 12 cell line, mouse KCs, murine monocyte/macrophage cell line RAW264.7	JNK↓, cytochrome P450 2E1↓, MDA↓, ALT↓, TNF-α↓, IL-6↓, IL-1β↓	Choi et al. (2019a)
GDS	C57BL/6 male mice, HepG2 cells	PPAR-γ↑, NF-κB↓, NLRP3↓, AST↓, ALT↓, AMPK↑, nuclear factor-erythroid 2 related factor 2/heme oxygenase 1↑, MDA↓, SOD↑	Mi et al. (2024)
GF_2_	C57BL/6J male and LXRα deficient mice, mouse BMDMs, mouse PHs	LXRα↓, IL-1β↓, TNF-α↓, IL-6↓	Kim et al. (2024a)
Ginsenosides	C57BL/6J male mice	NF-κB↓, ALT↓, AST↓, TNF-α↓, IL-1β↓, IL-6↓	Liang et al. (2021)
LFG	C57BL/6N male mice	ALT↓, AST↓, TNF-α↓	Nan et al. (2018)
Mc_1_	C57BL/6 male mice, HepG2 cells	GRP78↓, CHOP↓	Roh et al. (2020)
MFG	SD male rats	IgA↓	Zhao et al. (2021)
Panaxydol	C57BL/6 male mice, mouse BMDMs and KCs	TNF-α↓, lactate dehydrogenase↓, NLRP3↓, Caspase-1↓, IL-1β↓, IL-18↓	Kim et al. (2024b)
Rb_1_	C57BL/6J male mice, mouse 3T3-L1 fibroblast cells, HepG2 cells	ALT↓, AST↓	Li et al. (2022d)
Rc	C57BL/6J mice, mouse PHs	AST/ALT↓, SIRT6↑, TNF-α↓, IL-6↓, ROS↓	Yang et al. (2023b)
Rd	C57BL/6J mice, mouse PHs	SIRT6↑, NF-κB↓, TNF-α↓, IL-6↓, IL-1β↓, ROS↓, ALT↓, AST↓	Cui et al. (2023)
Rf	HepG2 cells	MMP9 gene	Chen et al. (2022)
RG	Thoroughbred riding horses, otsuka long-evans tokushima fatty rats, C57BL/6 mice, mouse PHs and KCs	ALT↓, AST↓, TNF-α↓, IL-1β↓, IL-12a↓, IL-12b↓, IL-17a↓, FA binding protein 4↓, JNK↓, natural killer cells activity↑, p38 mitogen-activated protein kinase↓	Hong et al. (2013), Jeong et al. (2018), Kim et al. (2019)
Rg_1_	SD male rats, HepG2 cells, C57BL/6J mice	ALT↓, AST↓, AKP↓, NF-κB↓, IL-6↓, IL-1β↓, IL-18↓, TNF-α↓, MCP-1↓, forkhead box O1 protein↑, SOD↑, catalase↑, glutathione↑, MDA↓, NLRP3↓, GPR78↓, CHOP↓, Caspase-12↓,ACOX-2↑, ATF3↓	Gu et al. (2021), Hou et al. (2022), Qi et al. (2020), Xiao et al. (2019), Xu et al. (2018), Xuan et al. (2015)
Rg_3_	C57BL/6 male mice, mouse 3T3-L1 pre-adipocyte cells	TNF-α↓, IL-1β↓, IL-6↓, IL-10↓	Lee et al. (2017)
Mixture Rh_1_ and Rg_2_	C57BL/6 male mice, mouse KCs and PHs, mouse hepatic stellate cells	ALT↓, TNF-α↓, CCL2↓, arginase 1↓, IL-10↓, IL-1β↓, ROS↓, NLRP3↓	Wang et al. (2021)
Rk_3_	C57BL/6J male mice, HepG2 cells, LX2 cell lines	ALT↓, AST↓, AKP↓, NLRP3↓, IL-6↓, IL-1β↓, TNF-α↓	Guo et al. (2023)
WGCM	C57BL/6 male mice	PGC-1α↑, nuclear respiratory factor 1↑, mitochondrial transcription factor A↑, ALT↓, MDA↓, glutamate dehydrogenase↑, glutathione↑	Lee et al. (2016)

Note: BMDMs, bone marrow-derived macrophages; KCs, kupffer cells; PHs, primary hepatocytes; SD, sprague dawley. ↓: inactivate or decrease; ↑: activate or increase.

#### 4.2.1 Inflammation

The core pathologic features of NASH and NAFLD encompass lipotoxicity-mediated hepatocellular injury and a chronic inflammatory cascade response. Controlling inflammation is one of the fundamental strategies for treatment (Wiering and Tacke, 2023). In this study, we systematically revealed the molecular mechanisms by which ginseng and its functional components modulate inflammatory signaling networks through multiple targets (Figure 3).

Lipotoxic substances induce hepatocyte damage, leading to abnormal release of alanine aminotransferase (ALT), aspartate aminotransferase (AST), alkaline phosphatase (AKP), and lactate dehydrogenase as a diagnostic indicator of the early stages of the disease. Ginseng and its functional components reduces serum levels of damage markers and this effect is directly related to hepatocyte protection (Kim M. Y. et al., 2024; Xuan et al., 2015). In addition, ginseng and its functional components inhibited the production of various inflammatory factors, such as tumor necrosis factor α (TNF-α), interleukin (IL) 6, IL-1β, IL-18, IL-10, IgA, arginase 1, and C-C motif chemokine ligand (CCL) 2. It also reduced the recruitment and activation of immune cells and improved the inflammatory microenvironment (Kim M. Y. et al., 2024; Wang et al., 2021; Zhao et al., 2021).

Lipid overaccumulation leads to aberrant activation of mammalian target of rapamycin C1, causing macrophage 1 polarisation and elevating levels of CCL2, CCL5, IL-1β, IL-6, inducible nitric oxide sythase and TNF-α. FRG reduced the levels of CCL2, CCL5, IL-1β, IL-6, inducible nitric oxide sythase and TNF-α by inhibiting aberrant activation of mTORC1, which reverses macrophage 1 type to macrophage 2 type polarization. It also increases the levels of the macrophage 2 markers CD163 and IL-10, achieving an anti-inflammatory-promoting repair dynamic balance (Choi S. Y. et al., 2019). RG significantly increased natural killer cells counts in otsuka long-evans tokushima fatty rats, improving their immunity (Hong et al., 2013). In addition, RG reduced the phosphorylation of p38 mitogen-activated protein kinase and decreased the secretion of the inflammatory cytokine IL-1β (Kim et al., 2019). RG attenuates downstream hepatic injury and inflammation by inhibiting the FA binding protein4/c-Jun N-terminal kinase (JNK) pathway and reduces the production of ALT, AST, TNF-α, IL-1β, IL-12a, IL-12b, and IL-17a (Jeong et al., 2018).

In the LXRs regulatory module, GF_2_ enhances the binding capacity of co-inhibitory factors to LXRα. Reduction of LXRα transcriptional activity improves intracellular lipid homeostasis and correspondingly reduces the number of immune cells and pro-inflammatory factors infiltrating the liver (Kim K. et al., 2024). The body assembles and activates nod-like receptor thermal protein domain associated protein 3 (NLRP3) inflammasome after sensing lipotoxic danger signals, which in turn induces the maturation of cysteine-containing aspartate-specific protease (Caspase) 1 and catalyses the production of pro-inflammatory cytokines IL-18 and IL-1β (Mridha et al., 2017; Yu et al., 2022). Ginseng and its functional components modulated NLRP3 inflammasome-mediated inflammation by blocking the activation of NLRP3 inflammasome and Caspase-1 (Kim M. Y. et al., 2024; Xu et al., 2018). Ginsenosides, Rg_1_ and Rd reduced the expression of pro-inflammatory factors by directly inhibiting the activation of NF-κB, which is an important nuclear transcriptional regulator of pro-inflammatory genes (Cui et al., 2023; Liang et al., 2021; Xiao et al., 2019; Xu et al., 2018). GDS inhibits nuclear translocation of NF-κB and downregulates transcription of key inflammatory factor genes through activation of AMPK/PPAR-γ (Mi et al., 2024). Another study found that Rc and Rd regulate the production of inflammatory factors TNF-α, IL-6 and IL-1β by increasing SIRT6 protein expression (Cui et al., 2023; Yang Z. et al., 2023). Rg_1_ specifically upregulates activating transcription factor (ATF) 3/acyl-coenzyme A oxidase (ACOX) 2 to alleviate NAFLD, and how Rg_1_ regulates NAFLD through these two genes is unknown (Gu et al., 2021). Leukocyte expression of active the MMP9 gene enhanced its ability to infiltrate during the inflammatory process, and Rf regulation of NAFLD inflammation may be related to the MMP9 gene (Chen et al., 2022).

#### 4.2.2 Oxidative stress

The occurrence of OS in the body indicates that the antioxidant system scavenges ROS at a slower rate than the rate of ROS production, which is an important factor in liver injury and NAFLD progression (Kumar et al., 2021). The tricarboxylic acid cycle transfers the generated NADH and FADH2 to oxygen *via* the electron transport chain, which generates large amounts of ATP and promotes ROS production (Chen et al., 2020). In addition non-electron transport chain sources of ROS, especially the compensatory acceleration of β-oxidation due to hepatic steatosis, appear to generate more ROS and cause OS (Clare et al., 2022). Ginseng improves OS by intervening in the following targets (Figure 3).

Rg_1_, WGCM, Rh_1_ and Rg_2_ reduced abnormally elevated levels of ROS and malondialdehyde (MDA) and increased the vigour of antioxidants such as superoxide dismutase (SOD), catalase, glutamate dehydrogenase and glutathione (Hou et al., 2022; Lee et al., 2016; Wang et al., 2021). Further studies revealed that GDS supplementation activated AMPK and further activated the nuclear factor-erythroid 2 related factor 2/heme oxygenase 1 signalling pathway, elevating the level of SOD and enhancing the antioxidant capacity of hepatocytes (Mi et al., 2024). Rg_1_ elevated nuclear FOXO1 protein levels and subsequently targeted to increase SOD and CAT expression and decrease MDA levels (Qi et al., 2020).

Rd repairs abnormal mitochondrial morphology, ameliorates REDOX disorder, and reduces ROS generation due to electron leakage from ETC (Cui et al., 2023). WGCM elevated the levels of mitochondrial biogenesis related factors, such as PGC-1α, nuclear respiratory factor 1 and mitochondrial transcription factor A, which inhibited mitochondrial OS and promoted mitochondrial biogenesis (Lee et al., 2016). SIRT6 protein attenuated the mitochondrial stress and ameliorated redox deficits in the organism, and ginseng Rc and Rd improved OS status in normal mice better than in SIRT6-deficient mice (Cui et al., 2023; Yang Z. et al., 2023). Rg_1_ attenuated OS injury-induced cellular senescence and mitigated hepatic OS by improving biological processes such as cellular matrix composition, membrane receptors, and cellular responses to the outside (Hou et al., 2022). GBCK25 inhibited cytochrome P450 2E1 expression and blocked the lipid peroxidation chain reaction, while down-regulating JNK phosphorylation and attenuating OS-associated cell injury (Choi N. et al., 2019).

#### 4.2.3 Endoplasmic reticulum stress

The accumulation of large amounts of FFAs in fatty liver promotes the production of lipotoxic substances causing ER structural disruption and decrease in number, and ERS occurs in hepatocytes in order to restore homeostasis of the internal environment (Lebeaupin et al., 2018; Lei et al., 2023). During this process, the ER will trigger the unfolded protein response (UPR) to restore the protein homeostasis of the ER, and if the ERS remains at a high level for a long period of time, the terminal UPR programme will trigger cell death (Senft and Ronai, 2015; Zhang et al., 2022). Lipotoxicity induced ERS-OS interaction network constitutes the core pathology axis. We outline the evidence that ginseng intervention in NAFLD is associated with ERS and discuss possible points of intervention (Figure 3).

ERS and OS are both adaptive responses to NAFLD in the early stages of the body, and become damaging factors when they exceed a certain limit, and they form a two-way positive feedback between them. Rg_1_ was found to achieve synergistic hepatoprotection through dual regulation of ERS-OS cross-talk (Xu et al., 2018). Furthermore, Rg_1_ significantly reduced OS-induced ERS marker levels, suah as CCAAT/enhancer binding protein (C/EBP) homologous protein (CHOP), glucose-regulated protein78 (GRP78) and Caspase-12. It also attenuated NLRP3 inflammasome-mediated associated inflammation. Binding of the ER chaperone protein GRP78 to unfolded or misfolded proteins will activate ERS sensors, including inositol-requiring enzyme 1α, PRKR-like ER kinase, and ATF6, which are the initiators of the major signalling cascade of the UPR and the survival mechanism of the ERS (Xu et al., 2018). UPR promotes restoration of ER homeostasis, but failure of restoration induces increased synthesis of pro-apoptotic proteins and triggers cell death. Mc_1_ reduced the expression levels of GRP78 and CHOP proteins, which inhibited ERS and UPR activation, and Mc_1_ has not been found to ameliorate ERS through activation of the AMPK signalling pathway (Roh et al., 2020).

### 4.3 Other ways

Recent reports have suggested that the pathogenesis of NAFLD is ‘multiple-hit’ and that the causative factors are unlikely to be the same in all patients, so that co-morbidities of multiple factors are necessary for the development of NAFLD (Wang et al., 2023). Many molecular pathways that promote the development of NAFLD, such as early IR, gut microbiota and metabolites, are involved in hepatocyte lipotoxic stress and injury, and further contribute to hepatocyte senescence and death, stellate cell activation and fibrosis (Wang et al., 2023). These pathways and mechanisms ultimately lead to the transition of NAFLD to NASH and possibly to end-stage liver disease (Loomba et al., 2021). The mechanisms by which ginseng affects these deficiencies are unclear and are corresponding summarised in this paper (Table 5).

**TABLE 5:** Summary of cellular senescence, cell death, IR and gut microbiota disorder by which ginseng ameliorates NAFLD.

Ginseng	Models	Cellular senescence, cell death, IR or gut microbiota disorder	Ref.
Aged ginseng	C57BL/6N male mice	Glucose-6-phosphatase↓, phosphoenolpyruvate carboxykinase↓, glucokinase↑, malic enzyme↓, glucose-6-phosphate dehydrogenase↓, FBG↓, fasting insulin↓	Chung et al. (2016)
FRG	C57BL/6N male mice, mouse PHs	Bcl-2↑, mitophagy↑	Choi et al. (2019b)
GDS	C57BL/6 male mice, HepG2 cells	FBG↓, glucose homeostasis↑	Mi et al. (2024)
GF_2_	C57BL/6J male and LXRα deficient mice, mouse BMDMs, mouse PHs	HOMA-IR↓	Kim et al. (2024a)
Ginsenosides	C57BL/6J male mice	ZO-1↑, occludin↑, gut microbiota disorder↓, F/B↓	Liang et al. (2021)
GSO	C57BL/6J mice, HepG2 cells, rat PHs	HOMA-IR↓, FBG↓, fasting insulin↓, CCL2↓, COL1↓	Kim et al. (2018)
GWDF	male SD rats	HOME-IR↓, gut microbiota disorder↓, F/B↓	Hua et al. (2021)
Mc_1_	C57BL/6 male mice, HepG2 cells	Caspase 3↓, Bax↓, Bcl-xL↑, JNK↓, TNF-α↓, IL-6↓, HOMA-IR↓	Roh et al. (2020)
MFG	SD male rats	F/B↓, gut microbiota disorder↓	Zhao et al. (2021)
Panaxydol	C57BL/6 male mice, mouse BMDMs and KCs	Lactate dehydrogenase↓, COL3↓, TIMP1↓, α-smooth muscle actin↓, transforming growth factor β↓	Kim et al. (2024b)
Rb_1_	C57BL/6J male mice, Mouse 3T3-L1 fibroblast cells, HepG2 cells	LC3 protein↑, p62 protein↓, miR-128↓, transcription factor EB↑, adiponectin↑, HOME-IR↓, FBG↓	Li et al. (2022d), Meng et al. (2023)
Rb_2_	C57BL/KsJ-Lepdb (db/db) mice, HepG2 cells	SIRT1↑, AMPK↑, p62 protein↓, LC3 protein↑, FBG↓	Huang et al. (2017)
Rc	C57BL/6J mice, mouse PHs	HOMA-IR↓, phosphoenolpyruvate carboxykinase↓, glucose-6-phosphatase↓, FBG↓, fasting insulin↓	Yang et al. (2023b)
Rd	C57BL/6J mice, mouse PHs	SIRT6↑, HOMA-IR↓, glucose intolerance↓	Cui et al. (2023)
Rf	HepG2 cells	BAZ1A gene	Chen et al. (2022)
RG	C57BL/6 male mice, mouse PHs and KCs	TIMP1↓, transforming growth factor β↓	Jeong et al. (2018)
Rg_1_	SD male rats, C57BL/6J mice, HHl-5 hepatocytes	Bax↓, Bcl-2↑, sphingosine-1-phosphate lyase 1↓, phospho-extracellular regulated protein kinases 1/2↑, p-AKT↑, tumor protein P53↓, CDKN2A↓, senescence-associated β-galactosidase↓, phospho-histone H2A.X↓, CDKN1A↓, STAT1↑, epidermal growth factor receptor↑	Hou et al. (2022), Li et al. (2022a), Qi et al. (2020), Xuan et al. (2015)
Mixture Rh_1_ and Rg_2_	C57BL/6 male mice, mouse KCs, mouse PHs, mouse hepatic stellate cells	TIMP1↓, COL1↓, COL3↓, lysyl oxidas↓, CCL2↓	Wang et al. (2021)
Rk_3_	C57BL/6J male mice, HepG2 cells, LX2 cell lines	PI3K/AKT↑, COL1↓, hypoxia-inducible factor 1α↓, TIMP1↓, F/B↓, gut microbiota disorder↓	Guo et al. (2023)

Note: BMDMs, bone marrow-derived macrophages; KCs, kupffer cells; PHs, primary hepatocytes; SD, sprague dawley. ↓: inactivate or decrease; ↑: activate or increase.

#### 4.3.1 Cellular senescence and death

**FIGURE 4:**
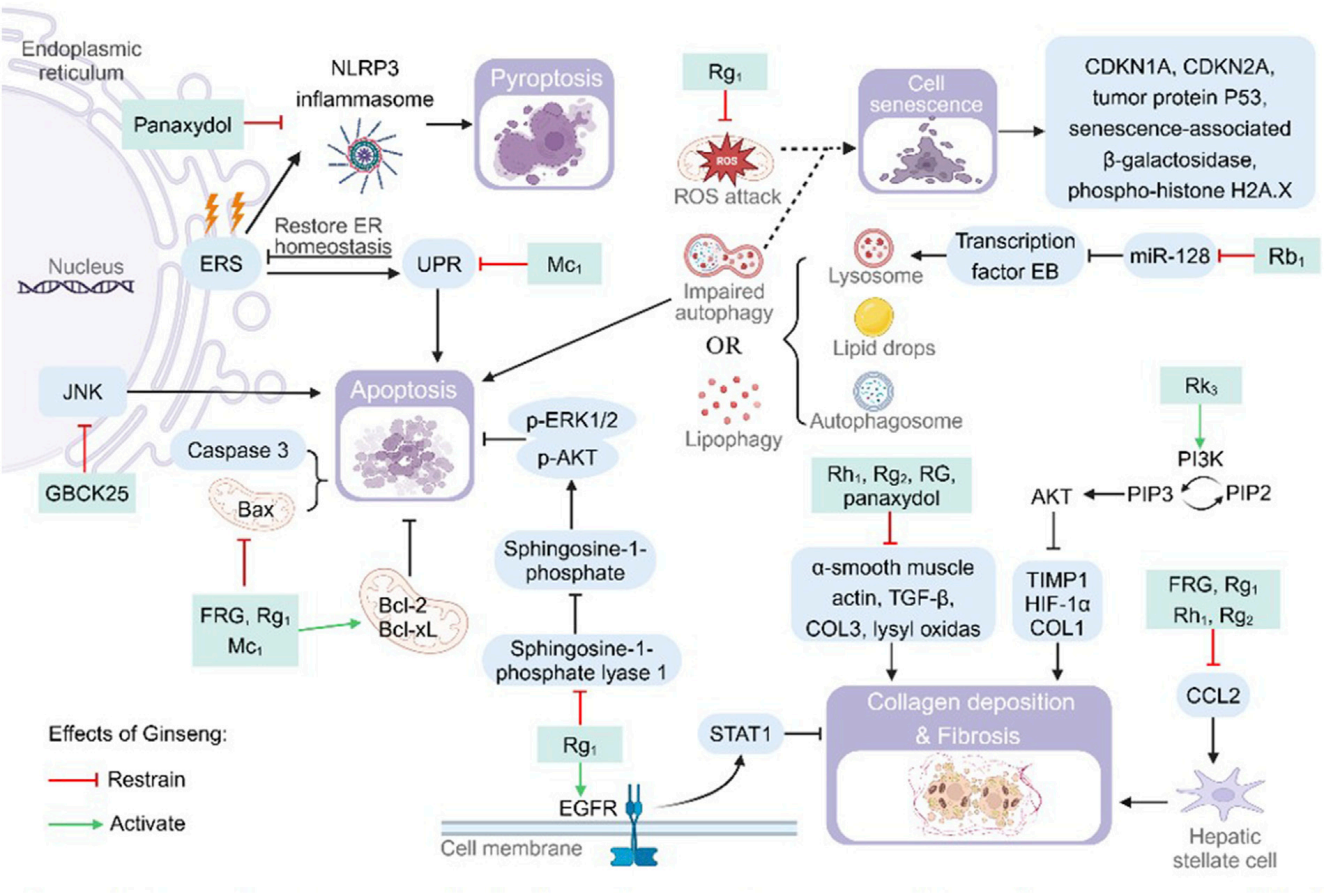
Pharmacological effects and molecular mechanisms of ginseng in reducing cellular senescence and death. Pathways or substances such as c-Jun N-terminal kinase (JNK), Bcl2-associated X (Bax), cysteine-containing aspartate-specific proteases (Caspase) 3 and sphingosine-1-phosphate lyase 1 have apoptosis-promoting effects, but ginseng and its characteristic metabolites inhibit the above processes. Endoplasmic reticulum stress (ERS) promotes cellular pyroptosis by upregulating nod-like receptor thermal protein domain associated protein 3 (NLRP3) inflammasome synthesis or apoptosis by promoting the unfolded protein response (UPR), but ginseng inhibits this process. Lysosomes, autophagosomes, and lipid droplets interact to cause lipophagy, and impairment of this process promotes cellular senescence. Ginseng attenuates cellular senescence by inhibiting miR-128 to increase the number of lysosomes or by inhibiting mitochondrial damage. Ginseng inhibits the expression of substances associated with collagen (COL) deposition and fibrosis, such as a-smooth muscle actin, transforming growth factor B (TGF-B), COL3 and lysyl oxidas, or inhibits liver fibrosis by activating the epidermal growth factor receptor (EGFR) and protein kinase B (PI3K/AKT) pathways. Bcl-2: B-cell lymphoma-2; Bcl-XL: B-cell lymphoma-extra large; CDKN: cyclin-dependent kinase inhibitor; ER: endoplasmic reticulum; HIF-1a: hypoxia-inducible factor 1a; p-AKT: phospho-protein kinase B; p-ERK: phospho-extracellular regulated protein kinases; PI3K: phosphatidylinositol-3-kinase; PIP2: phosphatidylinositol bisphosphate; PIP3: phosphatidylinositol trisphosphate; ROS: reactive oxygen species; STAT: signal transducer and activator of transcription; TIMP1: tissue inhibitor of metalloproteinase 1.

Hepatocyte death (apoptosis, pyroptosis, impaired autophagy) and fibrosis are key components of NAFLD progression to NASH (Shojaie et al., 2020). Hepatic injury factors such as fat deposition, inflammation, OS and ERS are involved in the pathological changes and accelerate the process of hepatocyte death and fibrosis. Ginseng significantly ameliorates a variety of cell death and aging-related pathological processes through the following multi-targeted mechanisms (Figure 4).

Upon stimulation of the organism from internal or external factors, pro-Caspase-3, the common downstream effector part of multiple apoptotic pathways, will be cleaved to the activator Caspase-3 and activate the cascade reaction of apoptosis (McComb et al., 2019). FRG, Rg_1_ and Mc_1_ significantly downregulated the expression levels of pro-apoptotic proteins Bcl-2-associated X (Bax) and Caspase-3, while up-regulating the expression levels of anti-apoptotic proteins B-cell lymphoma-2 (Bcl-2) and B-cell lymphoma-extra large (Bcl-xL) to reduce hepatocyte apoptosis (Choi S. Y. et al., 2019; Li G. et al., 2022; Roh et al., 2020). Supplementation of Mc_1_ lowered reduced CHOP and Bax activity. This process avoids prolonged high levels of ERS, which activates apoptosis induced by the terminal UPR program (Roh et al., 2020). Rg_1_ reduces sphingosine-1-phosphate cleavage by inhibiting sphingosine-1-phosphate lyase 1. Enhanced sphingosine-1-phosphate/p-AKT survival signaling in the liver and promoted hepatocyte survival (Li G. et al., 2022). GBCK25 reduced FFAs-induced steatotic apoptosis in hepatocytes by inhibiting the activation of the JNK pathway (Choi N. et al., 2019). Activation of the NLRP3 inflammasome promotes pro-Caspase-1 cleavage and activation. Activated Caspase-1 promotes both the maturation and release of IL-1β and IL-18, as well as being involved in the process of cellular pyroptosis. Panaxydol inhibited NLRP3 inflammatory vesicle activation (ASC oligomerization) and synthesis of lactate dehydrogenase (LDH), a marker of cellular cell death. The reduction of Caspase-1 activity and LDH content reversed the Caspase-1-dependent pathway of cellular cell death (Kim M. Y. et al., 2024). Lipophagy is the interaction of LC3 protein on the autophagosome membrane with envelope proteins on LDs to encapsulate LDs in autophagosomes for degradation. Abnormal lipophagy or degradation of autophagosomes (generation of p62 protein) will cause cell death. Rb_1_ and Rb_2_ increased autophagic vesicle survival by increasing LC3 protein and decreasing p62 protein content. Rb_2_ accelerated LD degradation by activating AMPK/SIRT1, upregulating LC3 protein and decreasing p62 protein (Huang et al., 2017; Meng et al., 2023). Rb_1_ enhances the transcription of transcription factor EB nuclear translocation and its downstream lysosome-associated genes by inhibiting miR-128. This resulted in increased lysosomal degradation of autophagic lipids and alleviated palmitic acid-induced autophagic flux block (Meng et al., 2023).

Additionally it has been proposed that although ginseng lowered Bax levels, it also elevated Bcl-2 during modeling of the disease. Because the Bax/Bcl-2 changes during treatment were slight, it is possible that the improvement in the course of Rg_1_ treatment for NAFLD was not cell death (Qi et al., 2020). In addition, impaired autophagy and free radical attack on mitochondria both contribute to cellular senescence. Rg_1_-treated mitochondria showed reduced swelling and vacuolization, more intact cytoplasmic matrix, and restoration of cellular autophagy. The expression of the key signals tumor protein P53, senescence-associated β-galactosidase, the nuclear damage marker phospho-histone H2A.X, and cyclin-dependent kinase inhibitor (CDKN) 1A/CDKN2A was decreased in the early stage of senescence (Hou et al., 2022; Qi et al., 2020). Validated by bioinformatic experiments and cellular experiments, Rf was found to inhibit aging-related phenotypes by affecting the BAZ1A gene (Chen et al., 2022). Hepatocyte injury or necrosis activates the body to repair damaged cells, and it causes hepatic fibroproliferation when repair persists. Panaxydol, Rg_1_, Rh_1_ and Rg_2_ was found to be effective in reducing the size of fibrotic areas, the number of inflammatory foci in the periportal and pericentric areas. Decreased the levels of liver fibrosis markers collagen (COL) 1, COL3, lysyl oxidas, tissue inhibitor of metalloproteinase 1 (TIMP1), α-smooth muscle actin, transforming growth factor β and hypoxia-inducible factor 1α (Kim M. Y. et al., 2024; Wang et al., 2021; Xu et al., 2018). In addition, Rk_3_ reduced the expression of hypoxia-inducible factor 1α, COL1 and hepatic TIMP1 through activation of the PI3K/AKT signaling pathway, and attenuated COL deposition and fibrosis in hepatocytes (Guo et al., 2023). FRG, Rg_1_, Rh_1_ and Rg_2_ inhibit high CCL2 expression. Reduced hepatic fibrosis and even cirrhosis formation by blocking CCL2 hepatic stellate cell activation (Choi S. Y. et al., 2019; Qi et al., 2020; Wang et al., 2021). Rg_1_ promotes the expression of downstream negative regulators of hepatic fibrosis and increases extracellular matrix degradation by regulating the epidermal growth factor receptor/STAT1 axis (Hou et al., 2022).

#### 4.3.2 Insulin resistance

**FIGURE 5:**
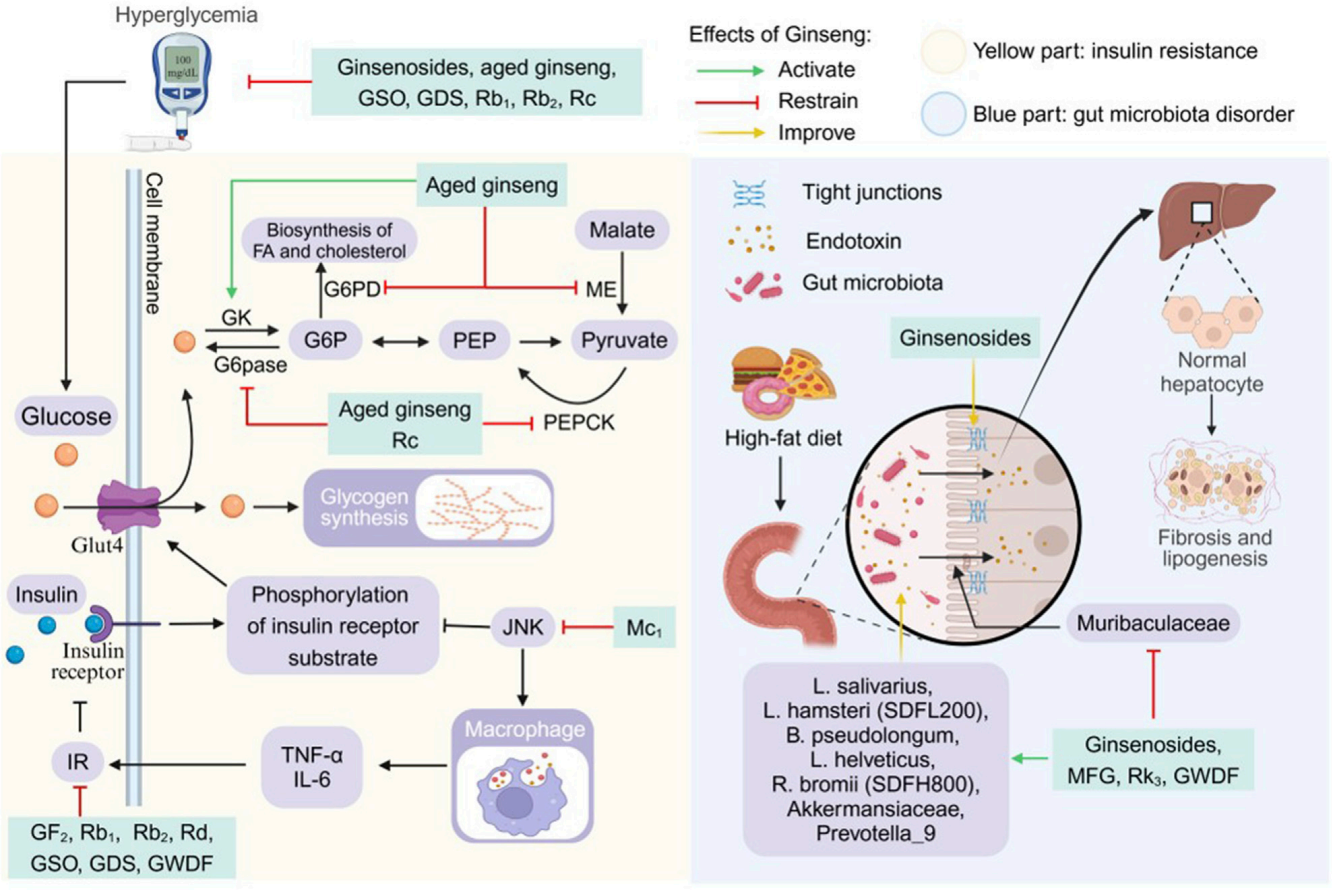
Pharmacological effects and molecular mechanisms of ginseng in ameliorating insulin resistance and gut microbiota disorders. Blue part: ginseng improved gut microbiota disorders and impaired intestinal vascular barrier induced by high-fat diet, and attenuated hepatic lipid deposition and hepatic fibrosis. Gray part: JNK reduces insulin sensitivity by inhibiting phosphorylation of insulin receptor substrateand promoting the production of inflammatory cytokines, but ginseng inhibits c-Jun N-terminal kinase (JNK) activation and insulin resistance (IR). Ginseng promotes the expression of glucokinase (GK), which activates glucose uptake and utilization, and inhibits glucose and lipid synthesis by inhibiting the activity of glucose-6- phosphatase (G6pase), phosphoenolpyruvate carboxykinase (PEPCK), malic enzyme (ME), and glucose-6-phosphate dehydrogenase (G6PD). FA: fatty acid; G6P: glucose-6-phosphate; GDS: ginseng diol saponin; Glu4: glucose transporter type 4; GSO: ginseng seed oil; GWDF: ginseng water-soluble dietary fiber; IL-6; interleukin 6; MFG: monascus ruber fermented ginseng; PEP: phosphoenolpyruvate; TNF-a: tumor necrosis factor a.

As a hub of systemic metabolic regulation, hepatic lipotoxicity injury (inflammation/OS/ERS) triggers systemic IR through multiple cascade reactions, which is manifested by (1) decreased glucose disposal in non-hepatic tissues (including adipose tissues and muscles). (2) dysregulation of lipolysis leading to aberrant lipid release. (3) impaired hepatic glycogen storage, which causes metabolic abnormalities and exacerbates systemic IR (Sakurai et al., 2021). In this study, we systematically elucidated the action network of ginseng in ameliorating disorders of glucolipid metabolism through multi-target synergism (Figure 5).

Ginseng and its functional components reduced fasting blood glucose (FBG) levels, fasting insulin and homeostatic model assessment for insulin resistance index (HOMA-IR) in animals of NAFLD disease model and improved glucose homeostasis, glucose tolerance and insulin tolerance in the organism (Cui et al., 2023; Kim K. et al., 2024; Li et al., 2022d). JNK elevates levels of TNF-α and IL-6 during obesity-driven macrophage activation, subsequently triggering IR. Mc_1_ inhibited the above process. In addition Mc_1_ restored insulin receptor substrate 1 tyrosine phosphorylation levels and increased insulin sensitivity in the process of driving down JNK phosphorylation levels (Roh et al., 2020). In regulating glucose metabolic activities, aged ginseng activates glucose uptake and utilization by decreasing insulin, FBG levels and increasing glucokinase expression. It also inhibits the synthesis of pyruvate, FAs and cholesterol by inhibiting malic enzyme and glucose-6-phosphate dehydrogenase enzyme activities (Chung et al., 2016). In addition both Rc and aged ginseng reduce the expression of glucose-6-phosphatase and phosphoenolpyruvate carboxykinase. This process reduces gluconeogenesis, decreases the body’s blood glucose level and improves glucose intolerance (Chung et al., 2016; Yang Z. et al., 2023). Rb_1_ improvement of IR and glucose tolerance is partially dependent on lipocalin. Specifically, it improves systemic glucose metabolism, insulin homeostasis, FA oxidation, and hepatic insulin sensitivity after activation of AMPK using lipocalin (Li et al., 2022d).

#### 4.3.3 Gut microbiota disorder

The microbial community in the human gastrointestinal tract has been shown to be involved in a variety of physiopathological processes in the gut, and a unique gut microbiome signature exists for NAFLD (Aron-Wisnewsky et al., 2020; Vallianou et al., 2021). The intestinal microecological disorder of NAFLD is characterized by the imbalance of “flora-intestinal-hepatic axis”, and the present study systematically reveals that ginseng can improve the metabolic abnormalities by regulating the homeostasis of intestinal flora in a multi-dimensional way (Figure 5) (Hsu and Schnabl, 2023).

High-fat diets increases intestinal permeability and exposes the liver to endotoxins, and a disturbed gut microbiota also disrupts the intestinal vascular barrier, enhancing intestinal permeability and bacterial lipopolysaccharide leakage into the circulation (Kobayashi et al., 2022; Mouries et al., 2019). Ginsenosides treatment increased the tight junction proteins ZO-1 and occludin levels in a dose-dependent manner, attenuating microecological dysregulation-mediated intestinal leakage and metabolic endotoxaemia (Liang et al., 2021). Qualitative and quantitative abnormalities of the gut microbiota are involved in the pathogenesis of NAFLD and NASH and promote hepatic lipogenesis and fibrosis (Hu et al., 2020; Kolodziejczyk et al., 2019). The study show that GWDF significantly increased the abundances of biomarkers such as L. salivarius, L. hamsteri (SDFL200), B. pseudolongum, *L. helveticus*, and R. bromii (SDFH800), and show a significant prebiotic effect. MFG and Rk_3_ selectively promoted colonisation by the probiotic bacteria Akkermansiaceae and Prevotella_9 and reduce the relative abundance of Muribaculaceae (Guo et al., 2023; Hua et al., 2021; Zhao et al., 2021). GWDF, ginsenosides, MFG and Rk_3_ had the effect of decreasing the Firmicutes and Bacteroidetes ratio, increasing the concentration of fecal short-chain FA, the abundance and diversity of intestinal flora species, and ameliorating the imbalance of gut microbiota induced by high-fat diets (Guo et al., 2023; Hua et al., 2021; Liang et al., 2021; Zhao et al., 2021). The above results demonstrated that ginseng has a three-in-one action model of “flora reconstruction, barrier repair and metabolic regulation”.

## 5 Conclusion and outlook

The burden caused by NAFLD is rising and treating NAFLD and its comorbidities is an important clinical challenge. Targeting lipid metabolism is gaining attention as a potential therapeutic target, and herbal medicine has been widely used as a complementary therapy for a long time. Ginseng and its functional components have hepatoprotective effects on patients and experimental animals, but ginseng itself has numerous components and complex targets and pathways, so finding safe and effective components and sorting out the mechanism of action deserve in-depth exploration. In this paper, we present a systematic review of the main features of ginseng in improving lipid metabolism in NAFLD through multiple components, pathways and targets.

In summary, ginseng and its functional components was found to help slow the progression of non-alcoholic fatty liver to NASH. Increased lipid uptake and synthesis, decreased catabolism and excretion, inflammation, ERS, OS, IR, cellular senescence, cell death and gut microbiota disorder are common pathogenic pathways. Modern studies have shown that ginseng and its functional components works by targeting AMPK, PPAR/PGC-1α, adipocytokine, FXR, LXR, SIRT, PI3K/AKT, JNK/insulin receptor substrate, sphingosine-1-phosphate/AKT/extracellular regulated protein kinases, nuclear factor-erythroid 2 related factor 2/heme oxygenase 1, PGC-1α/nuclear respiratory factor 1/mitochondrial transcription factor A, epidermal growth factor receptor, NLRP3 and NF-κB mechanisms to exert lipid-lowering and alleviate hepatic lipotoxicity. In addition, it can also affect the dynamics of lipid synthesis, oxidation and excretion by biometabolic pathways such as lipid *de novo* lipogenesis, β-oxidation, tricarboxylic acid cycle, gluconeogenesis, UPR, and mitochondrial biogenesis. This “network targeting-pharmacological” action characterizes the nature of NAFLD, which is characterized by multiple organ interactions and multiple pathologies.

At the level of mechanism research, we note that there is a cognitive bias in the current academic community that emphasizes on component mechanisms but not on systematic evaluation. Although ginseng has been shown to reduce hepatic cholesterol accumulation by activating FXR, it is puzzling that ginseng has been shown to promote cholesterol synthesis by up-regulating HMGCR expression (Li et al., 2020). It is worth noting that, compared with the compensatory metabolic escape phenomenon that often occurs with single-target chemical drugs. Ginsenosides may be more conducive to maintaining the long-term stability of glucose-lipid metabolism homeostasis by improving IR through the bi-directional regulation of insulin receptor substrate PI3K/AKT and JNK signaling (Roh et al., 2020). This paradoxical phenomenon suggests that there may be antagonistic effects among the complex components of Chinese medicines, and that relying solely on single component studies may result in misjudgment of the overall efficacy. Therefore, we strongly suggest that future studies should establish a three-dimensional evaluation system of “chemical component-biological effect-clinical phenotype,” and pay special attention to the key regulatory effects of non-saponin components such as ginseng polysaccharides and volatile oils on the intestinal flora-liver axis.

To date, ginseng is considered an edible and medicinal plant with a long history of medicinal use and a wide range of pharmacological effects. Ginseng and its functional components blocks the above pathogenesis of NAFLD through multi-components, multi-pathways, multi-targets and multi-levels, and has hepatoprotective effects on experimental animals. To summarise the relevant studies, the most relevant components of ginseng are ginsenosides, whose pharmacological mechanisms focus on the regulation of lipid metabolism and the subsequent reduction of lipotoxicity injury, and therefore we consider them to be the typical mechanisms of ginseng in the treatment of NAFLD. A study using network-based approaches to investigate the therapeutic effects and key mechanisms of ginseng revealed similar results (Kim Y. W. et al., 2024). Although the methods of these two studies are different, we adopt the attitude of seeking common ground while reserving differences, and believe that the results of the two studies can confirm and complement each other, verifying the rationality of ginseng in treating liver diseases. However, there are some slight differences between the two studies, such as network pharmacology found that regulation of protein function may be a key core target of ginseng in the treatment of liver-related diseases, but the experimental data on this aspect are slightly insufficient. From the perspective of clinical practice, we believe that ginseng has important application value because of its “both symptoms and root causes.” It can rapidly reduce hepatic TG deposition through acute activation of AMPK, and can also regulate the secretion of adipokines to realize the long-term benefit of metabolic memory. This dual-phase regulation advantage is extremely rare among existing chemical drugs, and we look forward to exploring it in more relevant studies in the future.

Taken together, these findings demonstrate the effectiveness of ginseng and its functional components in terms of key mechanisms against NAFLD. However, there are still some unknowns to be confirmed in this study. Firstly there are a few studies that showed discrepancies in the results, for example, ginseng reduced cholesterol transport by decreasing the activity of LDL receptor and increased the expression of HMGCR thereby increasing cholesterol synthesis (Li et al., 2020). We need more high-quality experiments to strengthen the level of evidence from controversial experiments. Secondly, in view of the shortcomings of the current quality control system, the current evaluation standard of saponin content as a single quality control index has seriously lagged behind the progress of basic research. We believe that a multidimensional quality control model should be constructed based on the correlation feature of “component-pathway-function,” which includes the assessment of bioavailability of metabolome and the detection of metabolic transformation ability of intestinal flora. For example, emphasizing ginseng’s absorption, disposition, metabolism, excretion and biosynthesis will remedy the shortcomings of the current quality control methods in order to better achieve the effect of comprehensive evaluation of the quality of TCM (Li X. et al., 2022). This innovative quality control concept may lead to a paradigm shift in TCM modernization research. Finally, there are still many important unexplored signalling molecules or pathways to be added in modern research. At the translational medicine level, for example, breakthroughs in the cutting-edge area of macrophage polarization regulation may lead to clinical benefits (Loomba et al., 2021). In genetic medicine, individualized intervention studies of ginseng for people with mutations in patatin-like phospholipase domain containing protein 3 may provide new ideas to address the therapeutic dilemma of genetically susceptible NAFLD (Park et al., 2023). It is also of interest that the regulation of the mitochondrial quality control network by the SIRT family has not yet been fully elucidated in ginseng studies, which may be an important bridge connecting lipotoxic injury to cellular aging mechanisms (Xu et al., 2024). However, the role of ginseng in this regard has been slightly underreported and more work needs to be done in these area.

Overall, the practice of ginseng functional components in the treatment of NAFLD is derived from the TCM theory, and its efficacy has been further verified by modern studies. We summarise the relevant studies and confirm that ginseng and functional components achieve therapeutic effects on NAFLD through multi-components, multi-targets, and multi-pathways. We believe that ginseng and its functional components play an ameliorative role in NAFLD, which has a complex pathogenesis, through multi-targets, and have significant pharmacological effects on NAFLD, reducing hepatic lipid accumulation by regulating lipid uptake and transport, increasing lipid catabolism, and decreasing lipid synthesis and other related mechanisms. In addition, ginseng ameliorates hepatic steatosis and hepatocyte injury by inhibiting lipotoxicity-related factors and pathways to attenuate hepatocyte inflammation, ERS, OS, cell death, cellular senescence and gut microbiota disorders. All these can promote the understanding and application of ginseng to compensate for the lack of NAFLD drugs and the development of green and natural medicines, so that more NAFLD patients can be helped.

## Glossary

ACC: acetyl-coenzyme A carboxylase
ACOX: acyl-coenzyme A oxidase
AKP: alkaline phosphatase
AKT: protein kinase B
ALT: alanine aminotransferase
AMPK: AMP-activated protein kinase
AST: aspartate aminotransferase
ATF: activating transcription factor
Bax: Bcl-2-associated X
Bcl-2: B-cell lymphoma-2
Bcl-XL: B-cell lymphoma-extra large
Caspase: cysteine-containing aspartate-specific protease
CCL: C-C motif chemokine ligand
CDKN: cyclin-dependent kinase inhibitor
CHOP: CCAAT/enhancer binding protein (C/EBP) homologous protein
ChREBP: carbohydrate responsive element-binding protein
COL: collagen
CPT: carnitine palmitoyl transferase
CYP7A1: cholesterol 7α-hydroxylase
ERS: endoplasmic reticulum stress
FA: fatty acid
FAS: fatty acid synthase
FBG: fasting blood-glucose
FFAs: free fatty acids
FG: fermented ginseng
FRG: fermentation of red ginseng with C. militaris
FXR: farnesoid X receptor
GDS: ginseng diol saponin
GRP78: glucose-regulated protein78
GSO: ginseng seed oil
GWDF: ginseng water-soluble dietary fiber
HDL-C: high density lipoprotein cholesterol
HMGCR: 3-hydroxy-3-methylglutaryl-coenzyme A reductase
HOMA-IR: insulin resistance index
IL: interleukin
IR: insulin resistance
JNK: c-Jun N-terminal kinase
LDL: low density lipid protein
LDL-C: low density lipoprotein cholesterol
LDs: lipid droplets
LFG: *lactobacillus* fermentum KP-3 fermented ginseng
LXR: liver X receptor
MDA: malondialdehyde
MFG: monascus ruber fermented ginseng
NAFLD: non-alcoholic fatty liver disease
NASH: non-alcoholic steatohepatitis
NF-κB: nuclear factor kappa-B
NLRP3: nod-like receptor thermal protein domain associated protein 3
Nrf2: nuclear factor-erythroid 2 related factor 2
PGC-1α: peroxisome proliferators-activated receptor γ co-activator α
PI3K: phosphatidylinositol-3-kinase
PPAR: peroxisome proliferator-activated receptor
PPD: protopanaxadiol
PPT: protopanaxatriol
RG: red ginseng
ROS: reactive oxygen species
SCD-1: stearoyl-coenzyme A desaturase-1
SIRT: sirtuins
SOD: superoxide dismutase
SREBP: sterol-regulatory element binding protein
STAT: signal transducer and activator of transcription
TC: total cholesterol
TCM: traditional Chinese medicine
TG: triglyceride
TIMP: tissue inhibitor of metalloproteinase 1
TNF-α: tumor necrosis factor α
UPR: unfolded protein response
WGCM: wild ginseng cambial meristematic cells
